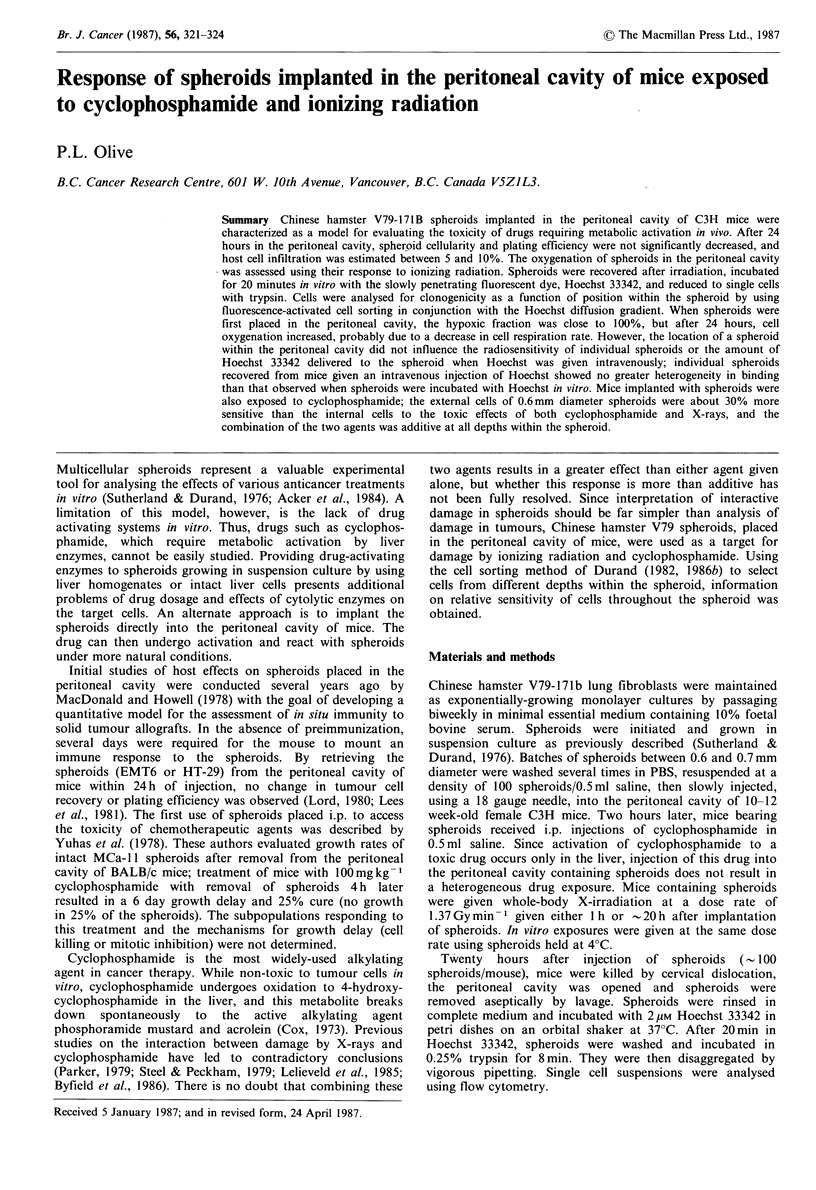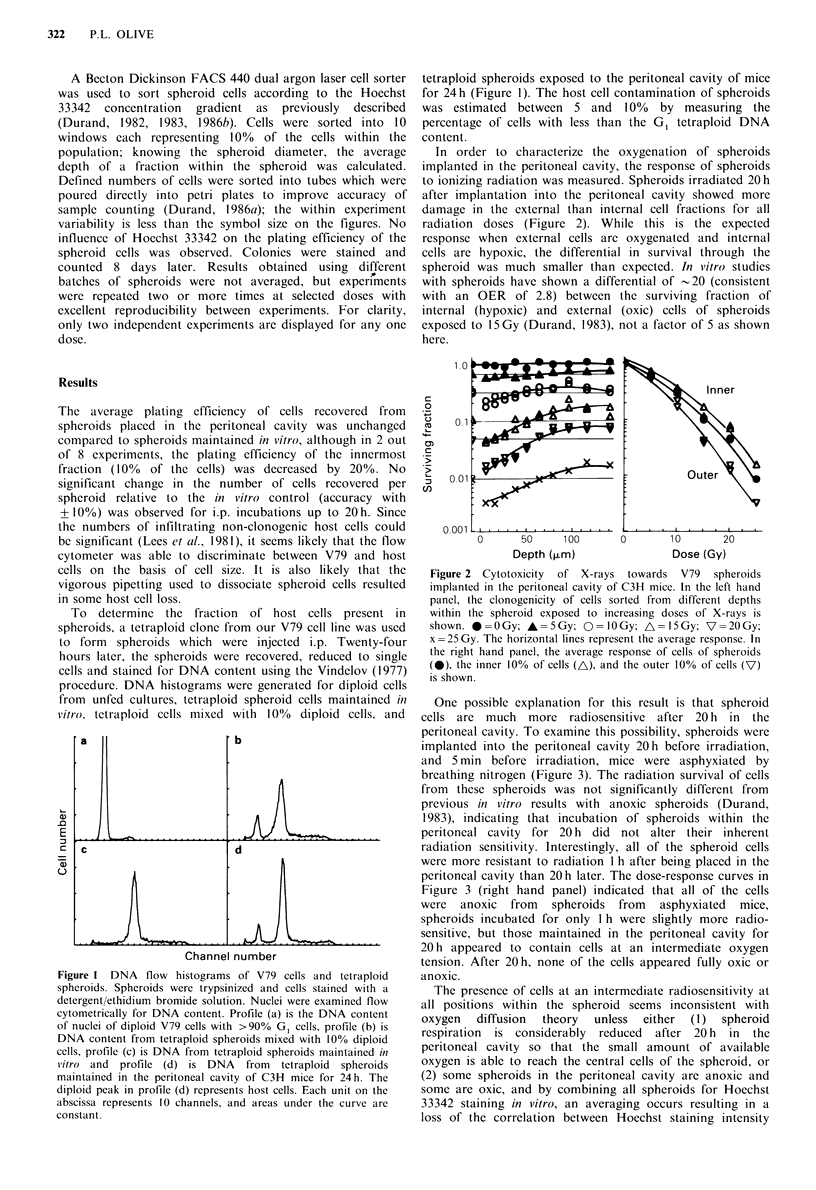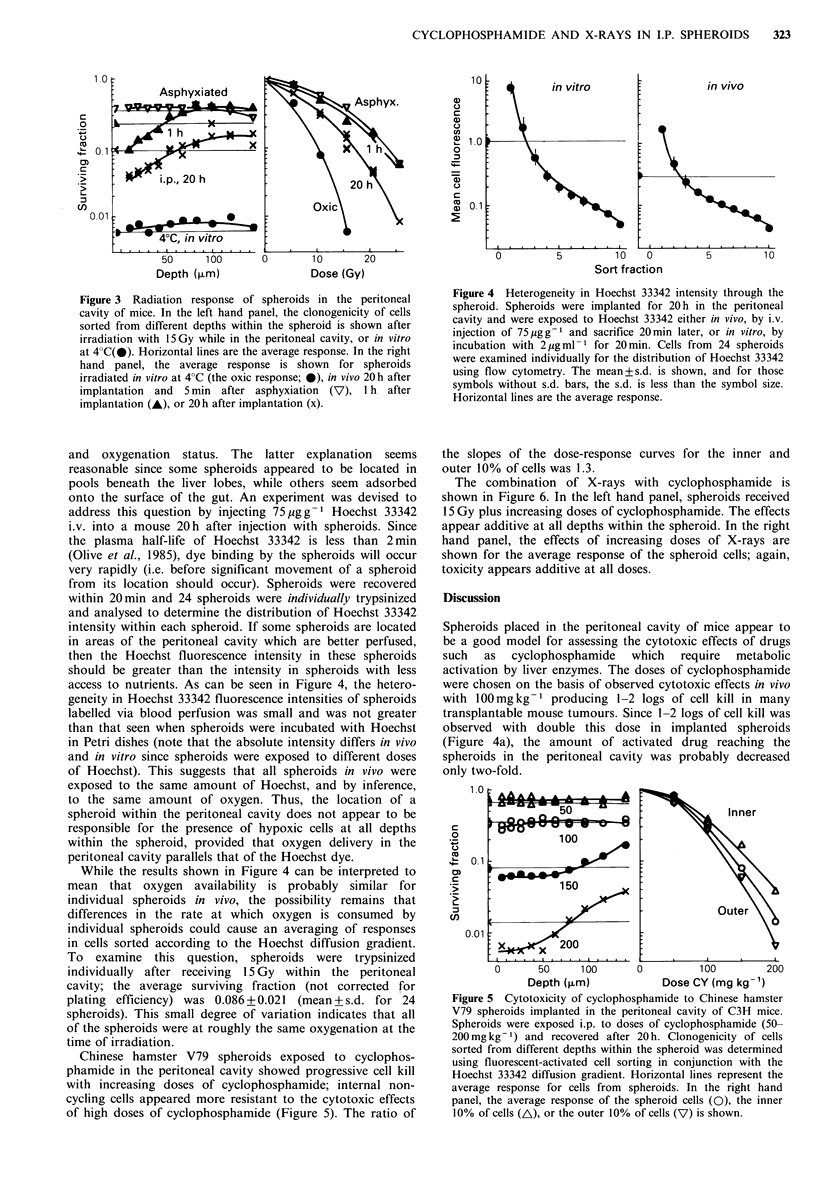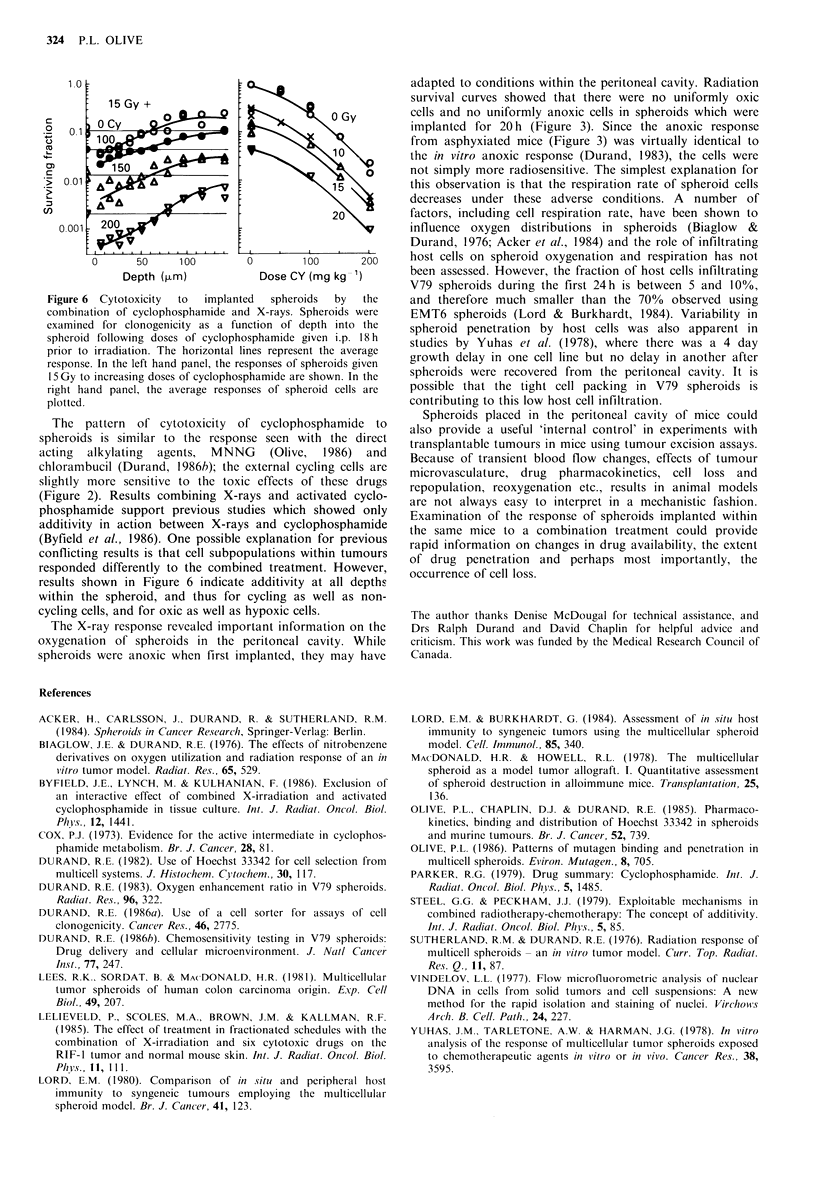# Response of spheroids implanted in the peritoneal cavity of mice exposed to cyclophosphamide and ionizing radiation.

**DOI:** 10.1038/bjc.1987.196

**Published:** 1987-09

**Authors:** P. L. Olive

**Affiliations:** B.C. Cancer Research Centre, Vancouver, Canada.

## Abstract

Chinese hamster V79-171B spheroids implanted in the peritoneal cavity of C3H mice were characterized as a model for evaluating the toxicity of drugs requiring metabolic activation in vivo. After 24 hours in the peritoneal cavity, spheroid cellularity and plating efficiency were not significantly decreased, and host cell infiltration was estimated between 5 and 10%. The oxygenation of spheroids in the peritoneal cavity was assessed using their response to ionizing radiation. Spheroids were recovered after irradiation, incubated for 20 minutes in vitro with the slowly penetrating fluorescent dye, Hoechst 33342, and reduced to single cells with trypsin. Cells were analysed for clonogenicity as a function of position within the spheroid by using fluorescence-activated cell sorting in conjunction with the Hoechst diffusion gradient. When spheroids were first placed in the peritoneal cavity, the hypoxic fraction was close to 100%, but after 24 hours, cell oxygenation increased, probably due to a decrease in cell respiration rate. However, the location of a spheroid within the peritoneal cavity did not influence the radiosensitivity of individual spheroids or the amount of Hoechst 33342 delivered to the spheroid when Hoechst was given intravenously; individual spheroids recovered from mice given an intravenous injection of Hoechst showed no greater heterogeneity in binding than that observed when spheroids were incubated with Hoechst in vitro. Mice implanted with spheroids were also exposed to cyclophosphamide; the external cells of 0.6 mm diameter spheroids were about 30% more sensitive than the internal cells to the toxic effects of both cyclophosphamide and X-rays, and the combination of the two agents was additive at all depths within the spheroid.


*Br. J. Cancer* (1987), **56**, 321–324 © The Macmillan Press Ltd., 1987

### Response of spheroids implanted in the peritoneal cavity of mice exposed to cyclophosphamide and ionizing radiation

P.L. Olive

*B.C. Cancer Research Centre, 601 W. 10th Avenue, Vancouver, B.C. Canada V5Z1L3.*

**Summary** Chinese hamster V79-171B spheroids implanted in the peritoneal cavity of C3H mice were characterized as a model for evaluating the toxicity of drugs requiring metabolic activation *in vivo*. After 24 hours in the peritoneal cavity, spheroid cellularity and plating efficiency were not significantly decreased, and host cell infiltration was estimated between 5 and 10%. The oxygenation of spheroids in the peritoneal cavity was assessed using their response to ionizing radiation. Spheroids were recovered after irradiation, incubated for 20 minutes *in vitro* with the slowly penetrating fluorescent dye, Hoechst 33342, and reduced to single cells with trypsin. Cells were analysed for clonogenicity as a function of position within the spheroid by using fluorescence-activated cell sorting in conjunction with the Hoechst diffusion gradient. When spheroids were first placed in the peritoneal cavity, the hypoxic fraction was close to 100%, but after 24 hours, cell oxygenation increased, probably due to a decrease in cell respiration rate. However, the location of a spheroid within the peritoneal cavity did not influence the radiosensitivity of individual spheroids or the amount of Hoechst 33342 delivered to the spheroid when Hoechst was given intravenously; individual spheroids recovered from mice given an intravenous injection of Hoechst showed no greater heterogeneity in binding than that observed when spheroids were incubated with Hoechst *in vitro*. Mice implanted with spheroids were also exposed to cyclophosphamide; the external cells of 0.6 mm diameter spheroids were about 30% more sensitive than the internal cells to the toxic effects of both cyclophosphamide and X-rays, and the combination of the two agents was additive at all depths within the spheroid.

Multicellular spheroids represent a valuable experimental tool for analysing the effects of various anticancer treatments *in vitro* (Sutherland & Durand, 1976; Acker *et al.*, 1984). A limitation of this model, however, is the lack of drug activating systems *in vitro*. Thus, drugs such as cyclophosphamide, which require metabolic activation by liver enzymes, cannot be easily studied. Providing drug-activating enzymes to spheroids growing in suspension culture by using liver homogenates or intact liver cells presents additional problems of drug dosage and effects of cytolytic enzymes on the target cells. An alternate approach is to implant the spheroids directly into the peritoneal cavity of mice. The drug can then undergo activation and react with spheroids under more natural conditions.

Initial studies of host effects on spheroids placed in the peritoneal cavity were conducted several years ago by MacDonald and Howell (1978) with the goal of developing a quantitative model for the assessment of *in situ* immunity to solid tumour allografts. In the absence of preimmunization, several days were required for the mouse to mount an immune response to the spheroids. By retrieving the spheroids (EMT6 or HT-29) from the peritoneal cavity of mice within 24 h of injection, no change in tumour cell recovery or plating efficiency was observed (Lord, 1980; Lees *et al.*, 1981). The first use of spheroids placed i.p. to access the toxicity of chemotherapeutic agents was described by Yuhas *et al.* (1978). These authors evaluated growth rates of intact MCa-11 spheroids after removal from the peritoneal cavity of BALB/c mice; treatment of mice with $100\,\mathrm{mg\,kg^{-1}}$ cyclophosphamide with removal of spheroids 4 h later resulted in a 6 day growth delay and 25% cure (no growth in 25% of the spheroids). The subpopulations responding to this treatment and the mechanisms for growth delay (cell killing or mitotic inhibition) were not determined.

Cyclophosphamide is the most widely-used alkylating agent in cancer therapy. While non-toxic to tumour cells *in vitro*, cyclophosphamide undergoes oxidation to 4-hydroxy-cyclophosphamide in the liver, and this metabolite breaks down spontaneously to the active alkylating agent phosphoramide mustard and acrolein (Cox, 1973). Previous studies on the interaction between damage by X-rays and cyclophosphamide have led to contradictory conclusions (Parker, 1979; Steel & Peckham, 1979; Lelieveld *et al.*, 1985; Byfield *et al.*, 1986). There is no doubt that combining these two agents results in a greater effect than either agent given alone, but whether this response is more than additive has not been fully resolved. Since interpretation of interactive damage in spheroids should be far simpler than analysis of damage in tumours, Chinese hamster V79 spheroids, placed in the peritoneal cavity of mice, were used as a target for damage by ionizing radiation and cyclophosphamide. Using the cell sorting method of Durand (1982, 1986*b*) to select cells from different depths within the spheroid, information on relative sensitivity of cells throughout the spheroid was obtained.

### Materials and methods

Chinese hamster V79-171b lung fibroblasts were maintained as exponentially-growing monolayer cultures by passaging biweekly in minimal essential medium containing 10% foetal bovine serum. Spheroids were initiated and grown in suspension culture as previously described (Sutherland & Durand, 1976). Batches of spheroids between 0.6 and 0.7 mm diameter were washed several times in PBS, resuspended at a density of 100 spheroids/0.5 ml saline, then slowly injected, using a 18 gauge needle, into the peritoneal cavity of 10–12 week-old female C3H mice. Two hours later, mice bearing spheroids received i.p. injections of cyclophosphamide in 0.5 ml saline. Since activation of cyclophosphamide to a toxic drug occurs only in the liver, injection of this drug into the peritoneal cavity containing spheroids does not result in a heterogeneous drug exposure. Mice containing spheroids were given whole-body X-irradiation at a dose rate of $1.37\,\mathrm{Gy\,min^{-1}}$ given either 1 h or ~20 h after implantation of spheroids. *In vitro* exposures were given at the same dose rate using spheroids held at 4°C.

Twenty hours after injection of spheroids (~100 spheroids/mouse), mice were killed by cervical dislocation, the peritoneal cavity was opened and spheroids were removed aseptically by lavage. Spheroids were rinsed in complete medium and incubated with 2 $\mu$M Hoechst 33342 in petri dishes on an orbital shaker at 37°C. After 20 min in Hoechst 33342, spheroids were washed and incubated in 0.25% trypsin for 8 min. They were then disaggregated by vigorous pipetting. Single cell suspensions were analysed using flow cytometry.

Received 5 January 1987; and in revised form, 24 April 1987.

A Becton Dickinson FACS 440 dual argon laser cell sorter was used to sort spheroid cells according to the Hoechst 33342 concentration gradient as previously described (Durand, 1982, 1983, 1986*b*). Cells were sorted into 10 windows each representing 10% of the cells within the population; knowing the spheroid diameter, the average depth of a fraction within the spheroid was calculated. Defined numbers of cells were sorted into tubes which were poured directly into petri plates to improve accuracy of sample counting (Durand, 1986*a*); the within experiment variability is less than the symbol size on the figures. No influence of Hoechst 33342 on the plating efficiency of the spheroid cells was observed. Colonies were stained and counted 8 days later. Results obtained using different batches of spheroids were not averaged, but experiments were repeated two or more times at selected doses with excellent reproducibility between experiments. For clarity, only two independent experiments are displayed for any one dose.

### Results

The average plating efficiency of cells recovered from spheroids placed in the peritoneal cavity was unchanged compared to spheroids maintained *in vitro*, although in 2 out of 8 experiments, the plating efficiency of the innermost fraction (10% of the cells) was decreased by 20%. No significant change in the number of cells recovered per spheroid relative to the *in vitro* control (accuracy with ±10%) was observed for i.p. incubations up to 20 h. Since the numbers of infiltrating non-clonogenic host cells could be significant (Lees *et al.*, 1981), it seems likely that the flow cytometer was able to discriminate between V79 and host cells on the basis of cell size. It is also likely that the vigorous pipetting used to dissociate spheroid cells resulted in some host cell loss.

To determine the fraction of host cells present in spheroids, a tetraploid clone from our V79 cell line was used to form spheroids which were injected i.p. Twenty-four hours later, the spheroids were recovered, reduced to single cells and stained for DNA content using the Vindelov (1977) procedure. DNA histograms were generated for diploid cells from unfed cultures, tetraploid spheroid cells maintained *in vitro*, tetraploid cells mixed with 10% diploid cells, and tetraploid spheroids exposed to the peritoneal cavity of mice for 24 h (Figure 1). The host cell contamination of spheroids was estimated between 5 and 10% by measuring the percentage of cells with less than the $G_1$ tetraploid DNA content.

**Figure 1** DNA flow histograms of V79 cells and tetraploid spheroids. Spheroids were trypsinized and cells stained with a detergent/ethidium bromide solution. Nuclei were examined flow cytometrically for DNA content. Profile (a) is the DNA content of nuclei of diploid V79 cells with >90% $G_1$ cells, profile (b) is DNA content from tetraploid spheroids mixed with 10% diploid cells, profile (c) is DNA from tetraploid spheroids maintained *in vitro* and profile (d) is DNA from tetraploid spheroids maintained in the peritoneal cavity of C3H mice for 24 h. The diploid peak in profile (d) represents host cells. Each unit on the abscissa represents 10 channels, and areas under the curve are constant.

In order to characterize the oxygenation of spheroids implanted in the peritoneal cavity, the response of spheroids to ionizing radiation was measured. Spheroids irradiated 20 h after implantation into the peritoneal cavity showed more damage in the external than internal cell fractions for all radiation doses (Figure 2). While this is the expected response when external cells are oxygenated and internal cells are hypoxic, the differential in survival through the spheroid was much smaller than expected. *In vitro* studies with spheroids have shown a differential of ~20 (consistent with an OER of 2.8) between the surviving fraction of internal (hypoxic) and external (oxic) cells of spheroids exposed to 15 Gy (Durand, 1983), not a factor of 5 as shown here.

**Figure 2** Cytotoxicity of X-rays towards V79 spheroids implanted in the peritoneal cavity of C3H mice. In the left hand panel, the clonogenicity of cells sorted from different depths within the spheroid exposed to increasing doses of X-rays is shown. ● = 0 Gy; ▲ = 5 Gy; ○ = 10 Gy; △ = 15 Gy; ▽ = 20 Gy; x = 25 Gy. The horizontal lines represent the average response. In the right hand panel, the average response of cells of spheroids (●), the inner 10% of cells (△), and the outer 10% of cells (▽) is shown.

One possible explanation for this result is that spheroid cells are much more radiosensitive after 20 h in the peritoneal cavity. To examine this possibility, spheroids were implanted into the peritoneal cavity 20 h before irradiation, and 5 min before irradiation, mice were asphyxiated by breathing nitrogen (Figure 3). The radiation survival of cells from these spheroids was not significantly different from previous *in vitro* results with anoxic spheroids (Durand, 1983), indicating that incubation of spheroids within the peritoneal cavity for 20 h did not alter their inherent radiation sensitivity. Interestingly, all of the spheroid cells were more resistant to radiation 1 h after being placed in the peritoneal cavity than 20 h later. The dose-response curves in Figure 3 (right hand panel) indicated that all of the cells were anoxic from spheroids from asphyxiated mice, spheroids incubated for only 1 h were slightly more radiosensitive, but those maintained in the peritoneal cavity for 20 h appeared to contain cells at an intermediate oxygen tension. After 20 h, none of the cells appeared fully oxic or anoxic.

The presence of cells at an intermediate radiosensitivity at all positions within the spheroid seems inconsistent with oxygen diffusion theory unless either (1) spheroid respiration is considerably reduced after 20 h in the peritoneal cavity so that the small amount of available oxygen is able to reach the central cells of the spheroid, or (2) some spheroids in the peritoneal cavity are anoxic and some are oxic, and by combining all spheroids for Hoechst 33342 staining *in vitro*, an averaging occurs resulting in a loss of the correlation between Hoechst staining intensity

**Figure 3** Radiation response of spheroids in the peritoneal cavity of mice. In the left hand panel, the clonogenicity of cells sorted from different depths within the spheroid is shown after irradiation with 15 Gy while in the peritoneal cavity, or *in vitro* at 4°C(●). Horizontal lines are the average response. In the right hand panel, the average response is shown for spheroids irradiated *in vitro* at 4°C (the oxic response; ●), *in vivo* 20 h after implantation and 5 min after asphyxiation (▽), 1 h after implantation (▲), or 20 h after implantation (x).

**Figure 4** Heterogeneity in Hoechst 33342 intensity through the spheroid. Spheroids were implanted for 20 h in the peritoneal cavity and were exposed to Hoechst 33342 either *in vivo*, by i.v. injection of 75 μg g$^{-1}$ and sacrifice 20 min later, or *in vitro*, by incubation with 2 μg ml$^{-1}$ for 20 min. Cells from 24 spheroids were examined individually for the distribution of Hoechst 33342 using flow cytometry. The mean ± s.d. is shown, and for those symbols without s.d. bars, the s.d. is less than the symbol size. Horizontal lines are the average response.

and oxygenation status. The latter explanation seems reasonable since some spheroids appeared to be located in pools beneath the liver lobes, while others seem adsorbed onto the surface of the gut. An experiment was devised to address this question by injecting 75 μg g$^{-1}$ Hoechst 33342 i.v. into a mouse 20 h after injection with spheroids. Since the plasma half-life of Hoechst 33342 is less than 2 min (Olive *et al.*, 1985), dye binding by the spheroids will occur very rapidly (i.e. before significant movement of a spheroid from its location should occur). Spheroids were recovered within 20 min and 24 spheroids were *individually* trypsinized and analysed to determine the distribution of Hoechst 33342 intensity within each spheroid. If some spheroids are located in areas of the peritoneal cavity which are better perfused, then the Hoechst fluorescence intensity in these spheroids should be greater than the intensity in spheroids with less access to nutrients. As can be seen in Figure 4, the heterogeneity in Hoechst 33342 fluorescence intensities of spheroids labelled via blood perfusion was small and was not greater than that seen when spheroids were incubated with Hoechst in Petri dishes (note that the absolute intensity differs *in vivo* and *in vitro* since spheroids were exposed to different doses of Hoechst). This suggests that all spheroids *in vivo* were exposed to the same amount of Hoechst, and by inference, to the same amount of oxygen. Thus, the location of a spheroid within the peritoneal cavity does not appear to be responsible for the presence of hypoxic cells at all depths within the spheroid, provided that oxygen delivery in the peritoneal cavity parallels that of the Hoechst dye.

While the results shown in Figure 4 can be interpreted to mean that oxygen availability is probably similar for individual spheroids *in vivo*, the possibility remains that differences in the rate at which oxygen is consumed by individual spheroids could cause an averaging of responses in cells sorted according to the Hoechst diffusion gradient. To examine this question, spheroids were trypsinized individually after receiving 15 Gy within the peritoneal cavity; the average surviving fraction (not corrected for plating efficiency) was 0.086 ± 0.021 (mean ± s.d. for 24 spheroids). This small degree of variation indicates that all of the spheroids were at roughly the same oxygenation at the time of irradiation.

Chinese hamster V79 spheroids exposed to cyclophosphamide in the peritoneal cavity showed progressive cell kill with increasing doses of cyclophosphamide; internal non-cycling cells appeared more resistant to the cytotoxic effects of high doses of cyclophosphamide (Figure 5). The ratio of the slopes of the dose-response curves for the inner and outer 10% of cells was 1.3.

The combination of X-rays with cyclophosphamide is shown in Figure 6. In the left hand panel, spheroids received 15 Gy plus increasing doses of cyclophosphamide. The effects appear additive at all depths within the spheroid. In the right hand panel, the effects of increasing doses of X-rays are shown for the average response of the spheroid cells; again, toxicity appears additive at all doses.

### Discussion

Spheroids placed in the peritoneal cavity of mice appear to be a good model for assessing the cytotoxic effects of drugs such as cyclophosphamide which require metabolic activation by liver enzymes. The doses of cyclophosphamide were chosen on the basis of observed cytotoxic effects *in vivo* with 100 mg kg$^{-1}$ producing 1–2 logs of cell kill in many transplantable mouse tumours. Since 1–2 logs of cell kill was observed with double this dose in implanted spheroids (Figure 4a), the amount of activated drug reaching the spheroids in the peritoneal cavity was probably decreased only two-fold.

**Figure 5** Cytotoxicity of cyclophosphamide to Chinese hamster V79 spheroids implanted in the peritoneal cavity of C3H mice. Spheroids were exposed i.p. to doses of cyclophosphamide (50–200 mg kg$^{-1}$) and recovered after 20 h. Clonogenicity of cells sorted from different depths within the spheroid was determined using fluorescent-activated cell sorting in conjunction with the Hoechst 33342 diffusion gradient. Horizontal lines represent the average response for cells from spheroids. In the right hand panel, the average response of the spheroid cells (○), the inner 10% of cells (△), or the outer 10% of cells (▽) is shown.

**Figure 6** Cytotoxicity to implanted spheroids by the combination of cyclophosphamide and X-rays. Spheroids were examined for clonogenicity as a function of depth into the spheroid following doses of cyclophosphamide given i.p. 18 h prior to irradiation. The horizontal lines represent the average response. In the left hand panel, the responses of spheroids given 15 Gy to increasing doses of cyclophosphamide are shown. In the right hand panel, the average responses of spheroid cells are plotted.

The pattern of cytotoxicity of cyclophosphamide to spheroids is similar to the response seen with the direct acting alkylating agents, MNNG (Olive, 1986) and chlorambucil (Durand, 1986*b*); the external cycling cells are slightly more sensitive to the toxic effects of these drugs (Figure 2). Results combining X-rays and activated cyclophosphamide support previous studies which showed only additivity in action between X-rays and cyclophosphamide (Byfield *et al.*, 1986). One possible explanation for previous conflicting results is that cell subpopulations within tumours responded differently to the combined treatment. However, results shown in Figure 6 indicate additivity at all depths within the spheroid, and thus for cycling as well as non-cycling cells, and for oxic as well as hypoxic cells.

The X-ray response revealed important information on the oxygenation of spheroids in the peritoneal cavity. While spheroids were anoxic when first implanted, they may have adapted to conditions within the peritoneal cavity. Radiation survival curves showed that there were no uniformly oxic cells and no uniformly anoxic cells in spheroids which were implanted for 20 h (Figure 3). Since the anoxic response from asphyxiated mice (Figure 3) was virtually identical to the *in vitro* anoxic response (Durand, 1983), the cells were not simply more radiosensitive. The simplest explanation for this observation is that the respiration rate of spheroid cells decreases under these adverse conditions. A number of factors, including cell respiration rate, have been shown to influence oxygen distributions in spheroids (Biaglow & Durand, 1976; Acker *et al.*, 1984) and the role of infiltrating host cells on spheroid oxygenation and respiration has not been assessed. However, the fraction of host cells infiltrating V79 spheroids during the first 24 h is between 5 and 10%, and therefore much smaller than the 70% observed using EMT6 spheroids (Lord & Burkhardt, 1984). Variability in spheroid penetration by host cells was also apparent in studies by Yuhas *et al.* (1978), where there was a 4 day growth delay in one cell line but no delay in another after spheroids were recovered from the peritoneal cavity. It is possible that the tight cell packing in V79 spheroids is contributing to this low host cell infiltration.

Spheroids placed in the peritoneal cavity of mice could also provide a useful 'internal control' in experiments with transplantable tumours in mice using tumour excision assays. Because of transient blood flow changes, effects of tumour microvasculature, drug pharmacokinetics, cell loss and repopulation, reoxygenation etc., results in animal models are not always easy to interpret in a mechanistic fashion. Examination of the response of spheroids implanted within the same mice to a combination treatment could provide rapid information on changes in drug availability, the extent of drug penetration and perhaps most importantly, the occurrence of cell loss.

The author thanks Denise McDougal for technical assistance, and Drs Ralph Durand and David Chaplin for helpful advice and criticism. This work was funded by the Medical Research Council of Canada.

### References

ACKER, H., CARLSSON, J., DURAND, R. & SUTHERLAND, R.M. (1984). *Spheroids in Cancer Research*, Springer-Verlag: Berlin.

BIAGLOW, J.E. & DURAND, R.E. (1976). The effects of nitrobenzene derivatives on oxygen utilization and radiation response of an *in vitro* tumor model. *Radiat. Res.*, **65**, 529.

BYFIELD, J.E., LYNCH, M. & KULHANIAN, F. (1986). Exclusion of an interactive effect of combined X-irradiation and activated cyclophosphamide in tissue culture. *Int. J. Radiat. Oncol. Biol. Phys.*, **12**, 1441.

COX, P.J. (1973). Evidence for the active intermediate in cyclophosphamide metabolism. *Br. J. Cancer*, **28**, 81.

DURAND, R.E. (1982). Use of Hoechst 33342 for cell selection from multicell systems. *J. Histochem. Cytochem.*, **30**, 117.

DURAND, R.E. (1983). Oxygen enhancement ratio in V79 spheroids. *Radiat. Res.*, **96**, 322.

DURAND, R.E. (1986*a*). Use of a cell sorter for assays of cell clonogenicity. *Cancer Res.*, **46**, 2775.

DURAND, R.E. (1986*b*). Chemosensitivity testing in V79 spheroids: Drug delivery and cellular microenvironment. *J. Natl Cancer Inst.*, **77**, 247.

LEES, R.K., SORDAT, B. & MACDONALD, H.R. (1981). Multicellular tumor spheroids of human colon carcinoma origin. *Exp. Cell Biol.*, **49**, 207.

LELIEVELD, P., SCOLES, M.A., BROWN, J.M. & KALLMAN, R.F. (1985). The effect of treatment in fractionated schedules with the combination of X-irradiation and six cytotoxic drugs on the RIF-1 tumor and normal mouse skin. *Int. J. Radiat. Oncol. Biol. Phys.*, **11**, 111.

LORD, E.M. (1980). Comparison of *in situ* and peripheral host immunity to syngeneic tumours employing the multicellular spheroid model. *Br. J. Cancer*, **41**, 123.

LORD, E.M. & BURKHARDT, G. (1984). Assessment of *in situ* host immunity to syngeneic tumors using the multicellular spheroid model. *Cell. Immunol.*, **85**, 340.

MACDONALD, H.R. & HOWELL, R.L. (1978). The multicellular spheroid as a model tumor allograft. I. Quantitative assessment of spheroid destruction in alloimmune mice. *Transplantation*, **25**, 136.

OLIVE, P.L., CHAPLIN, D.J. & DURAND, R.E. (1985). Pharmacokinetics, binding and distribution of Hoechst 33342 in spheroids and murine tumours. *Br. J. Cancer*, **52**, 739.

OLIVE, P.L. (1986). Patterns of mutagen binding and penetration in multicell spheroids. *Eviron. Mutagen.*, **8**, 705.

PARKER, R.G. (1979). Drug summary: Cyclophosphamide. *Int. J. Radiat. Oncol. Biol. Phys.*, **5**, 1485.

STEEL, G.G. & PECKHAM, J.J. (1979). Exploitable mechanisms in combined radiotherapy-chemotherapy: The concept of additivity. *Int. J. Radiat. Oncol. Biol. Phys.*, **5**, 85.

SUTHERLAND, R.M. & DURAND, R.E. (1976). Radiation response of multicell spheroids – an *in vitro* tumor model. *Curr. Top. Radiat. Res. Q.*, **11**, 87.

VINDELOV, L.L. (1977). Flow microfluorometric analysis of nuclear DNA in cells from solid tumors and cell suspensions: A new method for the rapid isolation and staining of nuclei. *Virchows Arch. B. Cell. Path.*, **24**, 227.

YUHAS, J.M., TARLETONE, A.W. & HARMAN, J.G. (1978). *In vitro* analysis of the response of multicellular tumor spheroids exposed to chemotherapeutic agents *in vitro* or *in vivo*. *Cancer Res.*, **38**, 3595.